# Supplementary figures and images for: Retrograde transport of Akt by a neuronal Rab5-APPL1 endosome
Source: Sci Rep. 2019 Feb 21;9:2433. doi: 10.1038/s41598-019-38637-0 (PMC6385319; doi:10.1038/s41598-019-38637-0)

Supp. Figure 2: Number of movement events of TrkB and APPL1 double positive endosomes

A.

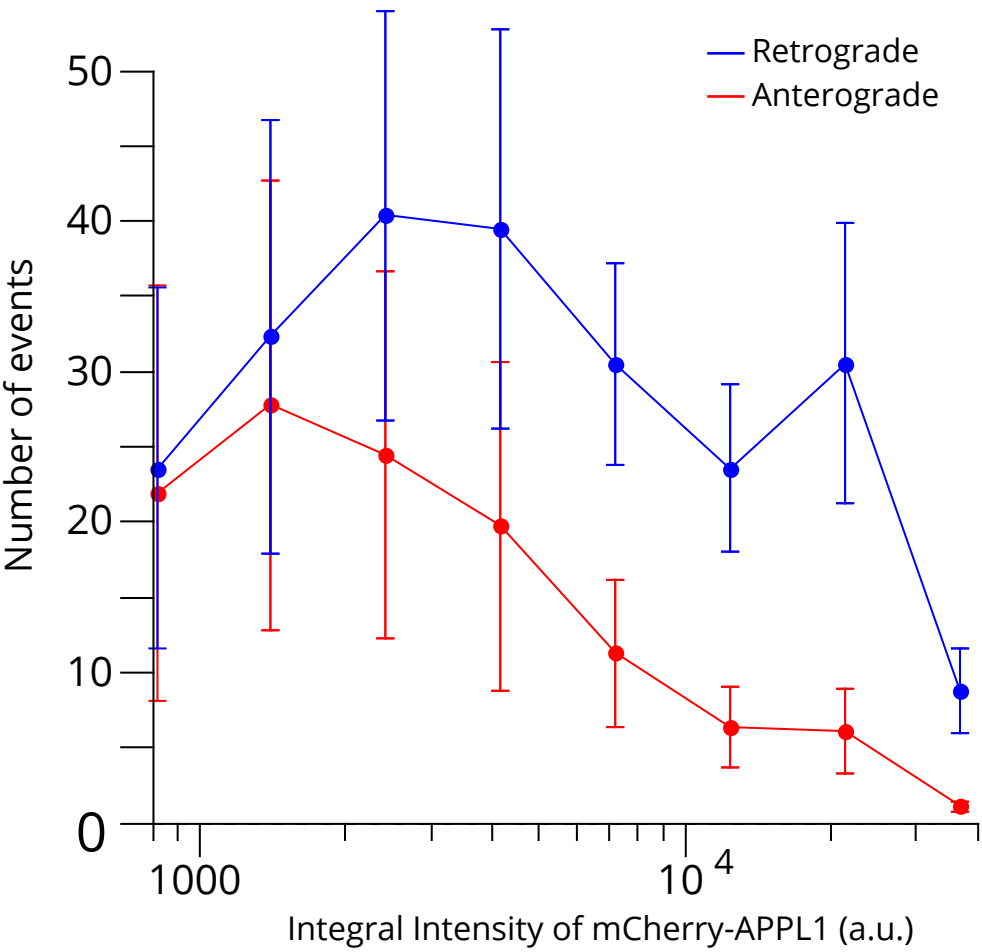

Supplement: Supplementary file 8 — Supp information [file 41598_2019_38637_MOESM8_ESM.pdf]
